# Measuring Digital Oral Health Literacy in the Social Media Era: Psychometric Validation of the Romanian Version of the Social Media Oral Health Literacy Questionnaire (SMOHLQ)

**DOI:** 10.3390/dj14040229

**Published:** 2026-04-13

**Authors:** Adina Oana Armencia, Andrei Nicolau, Laurian Lucian Francu, Galina Pancu, Roxana-Ionela Vasluianu, Georgiana Macovei, Alexandra Boloṣ, Dragos Catalin Ghica, Monica Mihaela Scutariu, Lucian Stefan Burlea

**Affiliations:** Grigore T. Popa University of Medicine and Pharmacy Iasi, 16 Universitatii Str., 700115 Iasi, Romania; adina.armencia@umfiasi.ro (A.O.A.); nicolau.andrei@umfiasi.ro (A.N.); laurianfrancu@yahoo.com (L.L.F.); galina.pancu@umfiasi.ro (G.P.); georgiana.macovei@umfiasi.ro (G.M.); alexandra.bolos@umfiasi.ro (A.B.); dragos.ghica@yahoo.ro (D.C.G.); mihaela.scutariu@umfiasi.ro (M.M.S.); lucianburlea@yahoo.com (L.S.B.)

**Keywords:** digital oral health literacy, social media, questionnaire validation, psychometric validation, SMOHLQ, dental students, health communication, eHealth literacy

## Abstract

**Background/Objectives**: Online environment and social media platforms have become major sources of health information, influencing behaviors and decision-making related to oral health. The Aim of This Study was to culturally adapt and psychometrically validate the Romanian version of the Social Media Oral Health Literacy Questionnaire (SMOHLQ) and to assess oral health literacy levels among dental students. **Material and Methods**: An observational cross-sectional study was conducted among 304 fourth- and fifth-year dental students. The Romanian version of the SMOHLQ underwent cultural adaptation and psychometric evaluation using descriptive statistics, exploratory factor analysis (principal axis factoring with oblimin rotation), Cronbach’s alpha coefficient, intraclass correlation coefficient (ICC), Pearson correlations, and ANOVA analysis. **Results**: Exploratory factor analysis confirmed a three-dimensional structure consisting of access and understanding, critical appraisal, and behavioral impact domains. Internal consistency was high for the overall scale (Cronbach’s α = 0.856) and good across subscales (α = 0.744–0.836). Pearson correlations showed significant associations between dimensions (r = 0.162–0.603, *p* < 0.001). ICC values indicated good score stability. ANOVA analyses revealed significant differences across demographic subgroups (*p* < 0.05). Mean scores were higher for cognitive dimensions (MS = 4.20–4.54) compared with behavioral impact (MS = 2.87). **Conclusions**: The Romanian version of the SMOHLQ demonstrated satisfactory psychometric properties, supporting its reliability and construct validity as a tool for assessing digital oral health literacy in the context of social media use.

## 1. Introduction

Oral health is a fundamental component of overall health, influencing daily functioning, social interactions, and quality of life. For this reason, maintaining optimal oral health is a constant priority for public health systems. Despite significant advances in the prevention and treatment of dental diseases, oral pathology continues to be among the most common health conditions/disorders worldwide and remains a major challenge for health policies. Dental caries and periodontal disease continue to have a considerable global impact, highlighting the decisive role of behavioral and socioeconomic factors in their onset and progression. According to the Global Burden of Disease study, approximately 3.74 billion cases of oral diseases were reported worldwide in 2021 [1].

In parallel with these developments, oral health research has gradually shifted from a predominantly treatment-focused biomedical model to a preventive and population-based approach that integrates cognitive, educational, and behavioral determinants of health. The rapid development of digital technologies has substantially changed the way medical information is accessed by individuals, with the online environment becoming one of the main sources of health information. This change has influenced both traditional health education strategies and the dynamics of the doctor–patient relationship, extending the concept of health literacy to the digital dimension, which involves the skills of identifying, critically evaluating, and applying information available online [2,3].

Information literacy is described as a set of skills that enable access, understanding, and use of health information necessary to maintain and promote health [4]. Low levels of health literacy are frequently associated with poor oral hygiene practices, increased prevalence of dental disease, and delayed use of dental services. Conversely, high levels of literacy are correlated with the adoption of preventive behaviors and more active patient involvement in therapeutic decision-making [4,5].

The digitalization of medical content has amplified the role of the online environment, especially among young adults, who frequently use digital platforms for information. Quick access to educational materials, professional recommendations and experiences shared by other users contributes to the formation of knowledge on the prevention and treatment of oral diseases [6,7,8]. In this context, digital health literacy can be considered an extension of traditional literacy, integrating navigation skills and critical evaluation of information from the online environment. Although eHealth and mHealth interventions can increase the information level and awareness, their impact on long-term behavior change remains variable between populations [9].

The high accessibility and interactive nature of social networks have transformed these platforms into an important channel for disseminating health information. Multimedia content distributed online promotes the rapid dissemination of messages on prevention and treatment and can stimulate interest in oral health. However, the lack of rigorous scientific validation mechanisms favors the circulation of incomplete or erroneous information [10]. Recent studies highlight a significant prevalence of misinformation in the oral health field, including the promotion of unrealistic therapeutic results or commercially motivated recommendations, all capable of influencing patient perception [11,12].

The literature shows that knowledge acquisition does not automatically lead to the adoption of health-promoting behaviors. Oral health practices result from the complex interaction of cognitive, attitudinal, and experiential factors, integrated into psychosocial models that explain how risk perception and past experiences influence oral hygiene behaviors and dental service addressability [13,14,15]. Simultaneous assessment of information literacy and actual behaviors provides a more comprehensive perspective on the determinants of oral health. Analysis of how individuals interact with digital health information can contribute to understanding the mechanisms through which informational factors influence preventive practices [4,5]. This issue is particularly relevant among young adults, characterized by intensive use of digital media and orientation towards online sources of information [7,16].

Thus, the use of social networks as a source of medical information generates a paradox specific to the contemporary digital environment: easy access to information can support health education but can simultaneously increase the risk of misinterpretation of medical content [17,18,19]. The effectiveness of digital educational tools depends largely on the ability of users to critically assess the source’s credibility and differentiate evidence-based information from promotional content [8,20].

As social media has become a major means of health information, the assessment of oral health literacy requires tools adapted to this context. Processing information distributed through social media requires additional skills, such as interpreting multimedia content, assessing the credibility of sources, and identifying commercial messages [21,22]. Platforms such as Facebook, YouTube, TikTok, Instagram, WhatsApp and Twitter are increasingly used for health education as well as for distributing multimedia information, thereby influencing user behavior [23]. The visual nature of dental treatments aligns with these platforms, thus becoming useful tools for patient education and strengthening preventive behaviors [8].

In this context, the Social Media Oral Health Literacy Questionnaire (SMOHLQ) was developed to evaluate oral health literacy in relation to social media use. At present, no standardized questionnaire specifically examines how oral health information available through social media is accessed, understood, critically appraised, and reflected in individual health-related behaviors. The instrument structure was guided by established theoretical frameworks of digital health literacy and oral health literacy, particularly the eHealth Literacy Scale (eHEALS) and the Digital Health Literacy Instrument (DHLI), which describe literacy as a set of competencies that include identifying relevant content, understanding its meaning, evaluating the reliability of sources, and applying knowledge when making health-related decisions. Based on these models, the items were formulated to reflect the particular features of social media environments, such as the predominance of multimedia content, variability in the quality of online materials, and the potential influence of digital content on oral health behaviors [24,25,26].

However, the use of a psychometric instrument in a different cultural context involves a rigorous process of linguistic adaptation and psychometric validation, as socio-cultural factors, digital literacy levels and social media usage patterns can influence the interpretation of the items. Currently, there is no adapted and validated version of the SMOHLQ for the Romanian population, which limits the standardized assessment of oral health literacy in the social media environment.

Thus, the primary aim of this study was to culturally adapt and assess the psychometric properties of the Romanian version of the Social Media Oral Health Literacy Questionnaire (SMOHLQ). A secondary aim was to describe the level of digital oral health literacy in the studied population and to explore potential differences across selected sociodemographic groups.

The study was based on the hypothesis that the Romanian version of the questionnaire would show good internal consistency and a factor structure consistent with existing models of digital health literacy. It was also hypothesized that the dimensions of the instrument would be positively associated, while reflecting distinct aspects of oral health literacy in the digital context. In addition, we explored whether the instrument could identify differences between population subgroups, providing further support for its validity.

## 2. Materials and Methods

### 2.1. Study Design

The study was conducted as an observational and cross-sectional study, with the aim of evaluating the psychometric properties of the questionnaire used to assess the level of oral health literacy. This design allowed for data collection at a single point in time, being suitable for analyzing the reliability and construct validity of the investigated instrument.

### 2.2. Participants and Selection Criteria

The study was conducted in two stages. In the first stage, the Romanian version of the SMOHLQ was obtained through a process of translation, back-translation, cultural adaptation, and evaluation by specialists, followed by verification of item clarity and comprehensibility. This preparatory stage took place before the questionnaire was administered to the target population and did not include data used in the final psychometric analysis.

Ethical approval (No. 718/22 February 2026) was obtained prior to the initiation of the main study and covered the recruitment of participants, the informed consent procedure, and the collection of questionnaire data. The cross-sectional study was carried out after obtaining ethical approval, in February 2026, among 4th- and 5th-year students of the Faculty of Dental Medicine, “Grigore T. Popa” University of Medicine and Pharmacy, Iași, Romania. After data collection was completed, the database was verified, statistical analyses were performed, and the manuscript was subsequently prepared.

The minimum required sample size was calculated using the calculation formula for cross-sectional studies, corresponding to a 95% confidence level (z = 1.96) and a 5% margin of error, reported to the target population of the study [27]. The calculation indicated a minimum required number of 205 participants. All eligible participants who voluntarily accepted participation were included in the study; the final sample consisted of 304 subjects.

The inclusion criteria in the study were students who:

- Are between 20 and 30 years old;
- Are enrolled in the year of study investigated at the time of the research;
- Have expressed their voluntary agreement to participate by signing the informed consent;
- Have the ability to understand and complete the questionnaires in Romanian;
- Have fully completed the applied assessment instruments.

The exclusion criteria from the study were:

- Incomplete or improperly completed questionnaires;
- Refusal to participate or withdrawal of informed consent;
- Presence of medical or psychological conditions that could have influenced the ability to understand and respond to the questionnaire items;
- Multiple participations or duplicate responses identified in the database;
- Lack of compliance in completing the assessment instruments.

The purpose and objectives of the study were clearly explained to the participants, highlighting the voluntary nature of participation and ensuring the confidentiality and anonymity of the data provided. The information necessary for conducting the research, including instructions on completing the questionnaires and calculating the final scores, was presented prior to data collection. After signing the informed consent, the participants completed the questionnaires included in the study.

Based on the main objective of the study, specific indicators were defined to guide the questionnaire evaluation and the selection of statistical analyses used in the psychometric assessment. The relationship between objectives, indicators and statistical methods is summarized in Table 1.

### 2.3. Research Instrument

To assess the level of digital literacy in oral health, the Social Media Oral Health Literacy Questionnaire (SMOHLQ) was used, whose purpose is to measure the ability of individuals to access, understand, critically evaluate and use information existing in the online environment related to oral health. In the specialized literature, the questionnaire was recently introduced as a tool for assessing the specific dimensions of digital literacy in oral health, being adapted for the context of online communication [28,29].

The questionnaire consists of 27 items, grouped into three main subdimensions, which reflect the stages of information processing on the internet:

Access and understanding (items 1–10): Assesses the ability of participants to identify, access and understand information about oral health available on digital platforms (Table 2).

Items 9 and 10 of the original questionnaire were not included in the dimensional analysis, as they are not part of the subscales used to calculate psychometric scores. Items 9 and 10 refer to social media use as a first source of information rather than to the ability to understand health information. For this reason, they were kept for descriptive analysis but were not included in the factorial model, as they do not directly reflect the cognitive dimension assessed by the scale.

Critical appraisal (items 11–21): Examines the ability to critically analyze content on the Internet, including assessing the credibility of sources and identifying commercial or potentially misleading information (Table 3).

Behavioral impact (items 22–27): Examines how social media information affects oral health behaviors and decisions (Table 4).

It should be noted that the items are formulated as self-reported statements and evaluated on a 5-point Likert scale; the degree of agreement of the respondents varies from 1 (“totally disagree”) to 5 (“totally agree”). Higher scores indicate a higher level of oral health literacy in the social media environment.

For each dimension, the average score is calculated, and the total score of the questionnaire results from the sum of the responses to all items. The multidimensional structure of the instrument allows for the distinct assessment of the cognitive (access and understanding), evaluative (critical analysis) and behavioral components of digital oral health literacy.

The translation and cultural adaptation of the questionnaire were carried out in accordance with international recommendations for the cross-cultural adaptation of psychometric instruments [30]. The original questionnaire was independently translated into Romanian by two bilingual translators (S.M. and R.G.), followed by the development of a consensual version. This version was subsequently back-translated into English by two independent translators (A.R. and H.S.), who did not have access to the original questionnaire. The back-translated version was compared with the original instrument in order to evaluate the semantic and conceptual items’ equivalence, resulting in the pre-final Romanian version of the instrument.

The pre-final version was further reviewed by specialists familiar with oral health research and health literacy terminology, with attention to the clarity and coherence and the appropriateness of the terminology used. The cultural adaptation process did not involve changes to the number of items, response options, or dimensional structure of the questionnaire and included only minor linguistic adjustments intended to ensure clarity and suitability for Romanian respondents.

Before being used in the main study, this version was reviewed informally by a small number of individuals similar to the target population in order to identify possible difficulties in understanding the formulation of the items. Before the main data collection, the pre-final version of the questionnaire was reviewed by 22 students similar to the target population to ensure that the enunciation of the items was clear and easy to understand. Based on their comments, minor linguistic adjustments were made to improve clarity, without changing the meaning of the original items. They were asked to indicate whether any questions were unclear or difficult to interpret. Based on these observations, minor phrasing adjustments were made where necessary, without modifying the meaning of the original items. This step was limited to improving linguistic clarity and did not involve formal data collection or inclusion of responses in the statistical analysis. The final Romanian version of the questionnaire was subsequently administered to the study participants after obtaining ethical approval.

### 2.4. Statistical Analysis and Psychometric Validation

The statistical analysis of the data was performed using the Statistical Package for the Social Sciences (ver. 26.0, IBM Corp., Armonk, NY, USA) program. The psychometric validation of the Romanian version of the Social Media Oral Health Literacy Questionnaire (SMOHLQ) was performed through a staged approach, which included the assessment of reliability and construct validity.

The validation procedure included the following stages:

- Descriptive statistical analysis, including the mean, standard deviation, minimum and maximum values, was used to characterize the distribution of scores for each dimension of the questionnaire.
- Internal consistency was evaluated by calculating Cronbach’s alpha coefficient for the overall questionnaire and for each dimension. Item–total correlations were examined in order to assess the contribution of each item to the corresponding dimension and to observe possible changes in the alpha coefficient in the case of item removal.
- Stability and consistency of the measurements were assessed using the intraclass correlation coefficient (ICC), calculated with a two-way mixed-effects model under a consistency definition for both individual and average measurements.
- Exploratory factor analysis (EFA) was employed to investigate the factor structure of the questionnaire and assess construct validity using the principal axis factoring method.
- The adequacy of the data for factor analysis was assessed using the Kaiser–Meyer–Olkin (KMO) index and the Bartlett test of sphericity.
- The optimal number of factors was determined based on the eigenvalues (eigenvalues > 1) and examination of the resulting factor structure.
- Oblique rotation (oblimin with Kaiser normalization) was applied to allow correlation of factors and obtain an interpretative factor structure.
- Internal construct validity was assessed by Pearson correlation coefficients between the questionnaire dimensions.
- Discriminant validity (known-groups validity) was analyzed using the unifactorial ANOVA test to compare SMOHLQ scores according to participant characteristics (e.g., gender and residential environment).

For all statistical analyses, a statistical significance threshold of *p* < 0.05 was considered significant.

## 3. Results

### 3.1. Sample Characteristics

The study included 304 participants enrolled in the fourth and fifth years at the Faculty of Dental Medicine, representing a population with a relatively homogeneous educational background and comparable exposure to digital sources of medical information. Females represented the majority of the sample (61.8%, n = 188), while males accounted for 38.2% (n = 116). The predominance of female participants is consistent with the demographic structure commonly reported in dental education programs, where female enrollment is generally higher.

Regarding the residence area, 56.9% of participants (n = 173) were from rural areas and 43.1% (n = 131) from urban areas. This distribution allowed the comparison of digital oral health literacy skills between individuals with potentially different levels of access to healthcare services, educational resources, and online medical information. Including participants from both urban and rural backgrounds provides a broader perspective on the variability of digital literacy skills and reduces the risk of bias associated with a single sociodemographic context (Table 5).

### 3.2. Factor Structure of the SMOHLQ

The adequacy of the data for factor analysis was evaluated using the Kaiser–Meyer–Olkin (KMO) index and Bartlett’s test of sphericity. The obtained KMO values ranged between 0.517 and 0.742, indicating acceptable sampling adequacy for exploratory factor analysis across all dimensions of the questionnaire. The lowest KMO value was observed for the critical appraisal dimension (0.517), corresponding to the minimum acceptable threshold, while the behavioral impact dimension showed the highest adequacy level (KMO = 0.742), indicating a good level of intercorrelations between items (Table 6).

Bartlett’s test of sphericity was statistically significant for all dimensions (*p* < 0.001), confirming the existence of sufficient correlations between items to justify factor extraction. These results support the suitability of the dataset for investigating the latent structure of the questionnaire.

Exploratory factor analysis using principal axis factoring with oblique rotation (Oblimin) confirmed the multidimensional structure of the instrument, consistent with the theoretical model underlying digital oral health literacy.

The dimension access and understanding showed a bidimensional structure, suggesting that the ability to search for oral health information online and the ability to interpret and understand that information represent related but distinguishable competencies. Factor loadings ranged between 0.525 and 0.759, indicating moderate to strong associations between items and the extracted factors (Table 7). One item (“I can quickly find information about dental symptoms or treatments online”) showed cross-loading on both extracted factors (0.525 and 0.464). This result suggests that the ability to rapidly identify online information may involve both search-related skills and the capacity to understand the retrieved content. Because these competencies are closely related in practice, the item was retained in the dimension, as its factor loadings were consistent with the theoretical structure of the questionnaire.

Similarly, the dimension critical appraisal presented a two-factor structure. The first factor included items related to the critical evaluation of online information, such as identifying exaggerated claims or questioning recommendations from non-professional sources, with factor loadings up to 0.956, suggesting strong relationships between items and the latent construct. The second factor grouped items associated with recognizing the credibility of sources and identifying commercial content, with loadings up to 0.826, indicating adequate representation of this dimension (Table 8). A similar situation was observed for the item “I can distinguish educational from advertising content”, which showed comparable loadings on both factors (0.437 and 0.449). This overlap may reflect the fact that identifying commercial intent often requires both evaluation of source credibility and interpretation of message content. Given the conceptual coherence of the item within the dimension, it was preserved in the factorial structure.

In contrast, the dimension behavioral impact demonstrated a unifactorial structure, with factor loadings ranging between 0.575 and 0.704, supporting the interpretation that the behavioral influence of social media information represents a relatively coherent construct (Table 9).

Overall, the factor analysis results support the theoretical assumption that digital oral health literacy involves sequential competencies related to accessing information, evaluating its credibility, and integrating it into health-related decision-making processes.

### 3.3. Reliability Analysis

Reliability analysis indicated a good level of internal consistency for the Romanian version of the SMOHLQ. The Cronbach’s alpha coefficient for the overall questionnaire was 0.856, indicating a high level of homogeneity between items and suggesting that the instrument consistently measures the underlying construct of digital oral health literacy.

At the dimensional level, Cronbach’s alpha coefficients ranged between 0.744 and 0.836. The access and understanding dimension demonstrated very good reliability (α = 0.836), indicating that the items included in this dimension measure a coherent construct related to the ability to obtain and comprehend oral health information in the digital environment.

For the critical appraisal dimension, the two factors identified through exploratory factor analysis showed good internal consistency, with α values of 0.784 and 0.744, respectively. These values suggest that the items included in this dimension adequately capture different aspects of critical evaluation, including the ability to assess the credibility of sources and to distinguish between scientific and promotional content.

The behavioral impact dimension also demonstrated good reliability (α = 0.786), indicating that the items consistently reflect the perceived influence of online information on oral health decisions and behaviors.

The intraclass correlation coefficient (ICC), calculated using a two-way mixed-effects model with a consistency definition, ranged between 0.786 and 0.812 for average measures. These values indicate a good level of agreement and support the stability of the scores obtained for each dimension. The concordance between Cronbach’s alpha and ICC values suggests that the instrument provides reliable and reproducible measurements of digital oral health literacy (Table 10). ICC values were calculated at the level of the overall dimension scores rather than separately for each factor identified through exploratory factor analysis. For the critical appraisal dimension, the reported ICC reflects the stability of the combined score, including items from both factors.

### 3.4. Descriptive Statistics of SMOHLQ Scores

Descriptive statistical analysis highlighted differences between the three dimensions of the questionnaire in terms of mean scores and variability of responses (Table 11).

The access and understanding dimension showed a high mean score (M = 4.20; SD = 0.36), with values ranging between 3.30 and 4.80. The relatively small standard deviation indicates a homogeneous distribution of responses, suggesting that most participants reported good abilities to search for and understand oral health information obtained from social media platforms.

The highest mean score was observed for the critical appraisal dimension (M = 4.54; SD = 0.40), with scores ranging between 3.55 and 5.00. These values indicate that participants generally reported a high level of ability to evaluate the credibility of online information, identify promotional content, and recognize potentially misleading messages. The relatively low variability of responses suggests a consistent level of critical evaluation skills within the study population.

In contrast, the behavioral impact dimension recorded a lower mean score (M = 2.87; SD = 0.69), with a wider range of values between 1.67 and 4.33. The higher variability observed for this dimension indicates differences between participants regarding the extent to which information obtained from social media influences oral health decisions and behaviors.

These results suggest that although participants demonstrate high levels of cognitive and evaluative digital literacy, the translation of information into behavioral changes appears less consistent, indicating that behavioral adaptation may depend on additional individual or contextual factors.

### 3.5. Construct Validity

Pearson correlation coefficients indicated statistically significant positive associations between all dimensions of the questionnaire, supporting the construct validity of the instrument (Table 12).

A moderate correlation was observed between access and understanding and critical appraisal (r = 0.603, *p* < 0.001), indicating that participants who reported better abilities to identify and understand oral health information also tended to demonstrate stronger skills in critically evaluating the credibility of online content.

The correlations between the cognitive dimensions and the behavioral impact dimension were weaker, with values of r = 0.270 (*p* < 0.001) and r = 0.162 (*p* = 0.005). These results indicate that although the dimensions are related, they represent distinct components of digital oral health literacy.

The lower magnitude of correlations involving the behavioral dimension suggests that a higher level of knowledge or critical evaluation ability does not necessarily lead to consistent changes in oral health behaviors, highlighting the multifactorial nature of behavioral decision-making.

### 3.6. Known-Groups Validity

Analysis of variance (ANOVA) was performed to evaluate the ability of the questionnaire to differentiate between population subgroups according to gender, residence area, and age (Table 13).

No statistically significant gender differences were observed for the access and understanding dimension (F = 0.406, *p* = 0.524), suggesting similar levels of ability to search and understand online oral health information among male and female participants.

However, statistically significant gender differences were identified for the critical appraisal dimension (F = 9.806, *p* = 0.002), indicating potential differences in the evaluation of online information between male and female participants. No statistically significant differences were observed for the behavioral impact dimension (F = 3.721, *p* = 0.055).

Comparisons according to residence area revealed statistically significant differences for both access and understanding (F = 47.540, *p* < 0.001) and critical appraisal (F = 7.796, *p* = 0.006), suggesting that participants from urban and rural environments differ in their ability to access and critically evaluate oral health information available online.

No statistically significant differences were observed for the behavioral impact dimension (F = 0.371, *p* = 0.543), indicating similar patterns regarding the influence of online information on oral health behaviors.

Age-related differences were statistically significant for all three dimensions of the questionnaire. For the access and understanding dimension, the analysis indicated significant differences between age groups (F = 21.578, *p* < 0.001). Similarly, the critical appraisal dimension showed statistically significant differences (F = 22.281, *p* < 0.001), suggesting that digital experience may influence the ability to evaluate online health information. The behavioral impact dimension also showed statistically significant differences according to age (F = 7.044, *p* = 0.001), indicating variability in the extent to which online information influences oral health behaviors across different age groups.

The mean SMOHLQ scores showed relatively small differences between the analyzed groups, but these were consistent with the results obtained from the ANOVA analysis.

According to gender, the scores for the access and understanding dimension were very similar between males (mean = 4.18, SD = 0.44) and females (mean = 4.21, SD = 0.30), indicating comparable levels of ability to identify and understand oral health information in the online environment. For the critical appraisal dimension, males showed slightly higher mean values (mean = 4.63, SD = 0.34) compared with females (mean = 4.48, SD = 0.42), a difference that corresponds to the statistically significant result observed in the ANOVA analysis. Regarding the behavioral impact dimension, females presented slightly higher scores (mean = 2.93, SD = 0.64) than males (mean = 2.77, SD = 0.76), suggesting a somewhat greater influence of online information on oral health-related behaviors.

In relation to residence area, participants from urban environments showed higher mean scores for both access and understanding (mean = 4.35, SD = 0.29) and critical appraisal (mean = 4.61, SD = 0.35) compared with those from rural areas (mean = 4.09, SD = 0.37 and mean = 4.49, SD = 0.42, respectively). This finding may indicate a slightly better ability among urban participants to identify and evaluate oral health information available online, possibly reflecting easier access to educational and digital resources. For the behavioral impact dimension, the mean values were very similar between the two groups (urban: mean = 2.84, SD = 0.76; rural: mean = 2.89, SD = 0.63), suggesting a comparable level of influence of online information on oral health behaviors.

Overall, the results indicate that group differences are more evident for the cognitive dimensions of digital oral health literacy (access and understanding and critical appraisal), while the behavioral dimension shows smaller variations across subgroups (Table 14).

These results support the discriminant validity of the instrument and indicate that digital oral health literacy skills may vary according to sociodemographic characteristics.

Overall, the results support the multidimensional structure, reliability, and construct validity of the Romanian version of the SMOHLQ. The instrument demonstrated good internal consistency, stable factor structure, and the ability to differentiate between population subgroups, supporting its suitability for assessing digital oral health literacy in the studied population.

## 4. Discussion

The results obtained in the study indicate that the instrument has adequate psychometric characteristics, demonstrating high internal consistency and a coherent factor structure, compatible with the theoretical model of digital health literacy. By applying a comprehensive psychometric approach, the study aimed to provide standardized evidence to support the use of SMOHLQ as a multidimensional instrument for assessing oral health literacy in the digital environment. Exploratory factor analysis confirmed the multidimensional organization of the questionnaire, highlighting three main domains, corresponding to the successive stages of interaction with online information: access and understanding of information, critical evaluation of digital content, and its behavioral impact. Overall, the results support the use of SMOHLQ as a valid instrument for assessing oral health literacy in the social media environment. The results are in line with previous studies conducted among dental students in Iași, which showed that oral health behaviors are influenced by cognitive and motivational factors, including attitudes toward health, perceived responsibility, and individual awareness of oral care. These findings suggest that the ability to understand and use health information plays an important role in supporting appropriate oral health behaviors, including in the context of information available online [31,32].

Exploratory factor analysis supported the multidimensional structure of the questionnaire, in line with the theoretical assumption that digital oral health literacy includes several related but distinct competencies. The KMO values and the statistically significant Bartlett test confirmed that the correlations between items were adequate for factor analysis. The access and understanding dimension showed a two-factor structure, suggesting that the identification of online information and its cognitive processing represent closely related but distinguishable processes. A similar pattern was observed for the critical appraisal dimension, which included two components reflecting both the active evaluation of digital content and the ability to judge the credibility of sources, including the recognition of commercial material. Some items showed cross-loadings between related factors, which may reflect the conceptual proximity between information search, interpretation, and evaluation processes in the digital environment. This finding is consistent with the multidimensional nature of digital health literacy, where competencies may partially overlap rather than function as completely independent constructs. In contrast, the behavioral impact dimension showed a unifactorial structure, indicating that the influence of online information on behavior may represent a more unified construct.

The factorial structure identified through exploratory analysis should be interpreted with caution, as confirmatory factor analysis was not performed in the present study. Although exploratory factor analysis is appropriate in the early stages of instrument validation, confirmatory approaches are generally required in order to statistically test the correspondence between the proposed theoretical model and the observed data. For this reason, the results should be regarded as providing initial support for the factorial structure of the Romanian version of the SMOHLQ rather than definitive confirmation of the model. Further evaluation on independent samples would allow a more rigorous examination of the stability of the proposed dimensions.

The KMO value for the critical appraisal dimension (0.517) was close to the minimum acceptable threshold, suggesting a moderate level of shared variance among the items included in this subscale. This result may reflect the nature of evaluating online health information, which involves several related, but not identical, skills, such as assessing source credibility, identifying commercial intent, and interpreting digital content. Because these competencies do not completely overlap, a lower level of factor homogeneity may be expected. Future studies may consider refining the wording of certain items or including additional items in order to improve the factorial performance of this dimension.

In addition, item 7 from the access and understanding dimension (“I can quickly find information about dental symptoms or treatments online”) showed cross-loading on both extracted factors. This result suggests that the ability to rapidly locate information online may involve both search-related skills and comprehension abilities. In digital contexts, these processes often occur simultaneously, making it difficult to fully separate them at the psychometric level.

Although the item was retained due to its conceptual relevance and acceptable loading values, these findings should be considered as psychometric limitations of the current version of the instrument. Future studies may explore minor adjustments in item wording or the inclusion of additional items in order to improve the internal coherence of the critical appraisal dimension and to further clarify the distinction between closely related competencies.

The relatively low KMO value observed for the critical appraisal dimension may reflect the heterogeneous nature of the competencies involved in evaluating online health information. Assessing credibility, identifying commercial intent and interpreting digital content involve related but not identical cognitive processes, which may reduce the degree of shared variance between items. In addition, the presence of cross-loading items suggests a partial overlap between competencies related to searching, understanding and evaluating online information. In real-life digital contexts, these processes are often interconnected, which may explain the difficulty in clearly separating them at the psychometric level. Although the retained items showed acceptable loadings and conceptual relevance, minor refinements of item wording or alternative factorial specifications could be explored in future studies in order to improve the separation between closely related dimensions.

These results are consistent with contemporary models of digital health literacy, which describe a progressive process that includes accessing information, evaluating it, and subsequently integrating it into health behaviors. Thus, the study conducted by van der Vaart R et al. on the development of the Digital Health Literacy Instrument (DHLI) demonstrated that digital literacy includes a wide range of complementary skills, organized into distinct dimensions, reflecting the successive stages of interaction with digital information (from identifying information to influencing health behaviors) [26,33].

The data available in the specialized literature highlight the fact that social media platforms facilitate the accessibility of information, contribute to the explanation of medical content, and support the application of information in oral health behaviors and decisions. At the same time, the difficulties related to assessing the credibility of online information and the risk of misinformation justify the existence of distinct critical analysis skills, which conceptually support the separation of the dimensions identified through factor analysis [28,34,35].

The reliability analysis indicated that the Romanian version of the SMOHLQ shows a satisfactory level of internal consistency. The values of Cronbach’s alpha obtained for the overall scale and for each dimension suggest that the items measure related aspects of digital oral health literacy and present an acceptable level of homogeneity. These results indicate that the items included in the questionnaire are consistent with each other and contribute to the assessment of the same underlying construct.

The item–total correlations showed that each item contributed appropriately to its corresponding dimension, without evidence that the removal of any item would substantially improve the internal consistency of the scale. This finding supports the coherence of the questionnaire structure and indicates that the items are relevant for the evaluation of the investigated construct.

The intraclass correlation coefficient (ICC) also indicated a good level of stability and consistency of the measurements. The obtained ICC values suggest that the instrument provides reproducible results and that the scores show an adequate level of stability for use in research settings.

Overall, the reliability indicators obtained in this study support the use of the Romanian version of the SMOHLQ as a reliable tool for assessing digital oral health literacy in the studied population.

The significant positive correlations identified between the dimensions of the questionnaire support the construct validity of the instrument. The moderate association between access to information and critical evaluation indicates that people able to effectively identify online information tend to develop superior skills in analyzing its correctness.

In contrast, the lower correlations between cognitive dimensions and the behavioral component suggest that a high level of information literacy does not automatically lead to behavioral changes. This result reflects the difference between knowledge and the effective adoption of health-promoting behaviors.

ANOVA analyses demonstrated the questionnaire’s ability to differentiate population subgroups, confirming the discriminant validity of the instrument. The differences observed between participants from urban and rural areas suggest the influence of access to educational and informational resources on the development of digital literacy skills.

Thus, mobile applications used for health monitoring, educational elements on oral hygiene methods, and information on the importance of dispensary visits existing on social media platforms allow for informed decision-making regarding oral health and facilitate the management of chronic diseases [9,36,37].

The age-related differences identified for all dimensions of the questionnaire indicate that digital experience and progressive exposure to online information may contribute to the development of skills in evaluating and using health information. Also, the variations observed by gender for the critical appraisal dimension may reflect differences in the way of processing digital information.

These results highlight the importance of sociodemographic factors in shaping digital literacy in oral health.

The results obtained show that the behavioral impact of digital information is also influenced by additional factors, such as individual motivation or social context. Evidence from interdisciplinary research indicates that repeated functional behaviors and environmental exposures may lead to adaptive changes within the stomatognathic system, highlighting the complex relationship between behavior and oral health outcomes [38]. In this context, effective dentist–patient communication plays a central role in motivating individuals to adopt and maintain appropriate oral health behaviors, as behavioral change largely depends on how health information is understood and internalized by patients. Moreover, studies conducted among dental students have demonstrated that oral health behaviors are strongly influenced by cognitive–perceptual factors such as self-efficacy, perceived health responsibility, and behavioral intentions [31].

These findings may also be interpreted from a biopsychosocial perspective, suggesting that oral health outcomes are influenced not only by clinical factors but also by behavioral and psychosocial aspects. The lower scores observed for the behavioral impact dimension indicate that the ability to access and critically evaluate information does not always translate into consistent changes in oral health practices. This difference may reflect the influence of individual motivation, previous experiences, attitudes toward prevention, and perceived relevance of the information [31,32]. In addition, social context and habitual behaviors may affect the extent to which information obtained online is integrated into daily oral care decisions. In this context, oral health literacy may facilitate understanding and evaluation of health information, but the embracement of health-promoting behaviors is likely influenced by a combination of cognitive, psychological, and social factors. These aspects may explain why relatively high levels of access and critical appraisal are not always accompanied by equally high behavioral impact scores [39]. The integration of social media platforms and digital solutions into daily oral care practices may contribute to preventing the progression of oral diseases and reducing associated costs. Recent studies have shown that tele-dentistry and digital tools enhance access to oral healthcare by enabling remote consultations, patient education, and continuous monitoring of treatment outcomes. These digital approaches support patient engagement and contribute to improved oral health literacy by facilitating better understanding and use of health information in preventive and therapeutic decision-making [39]. However, despite technological advances in dentistry, significant disparities persist in disadvantaged populations, where limited access to reliable health information and insufficient digital skills continue to affect oral health outcomes [40].

Previous studies show that a substantial proportion of adults experience difficulties understanding and using health-related information. Limited health literacy has been associated with problems in interpreting medical recommendations and identifying reliable sources of information. As the internet and social media have become common sources of health information, these difficulties may also influence how individuals search for and evaluate oral health information in online environments [41,42]. Recent systematic reviews also report considerable variability in health literacy levels across populations, with many individuals facing challenges in understanding medical information and treatment recommendations. Lower health literacy has been consistently associated with poorer health outcomes, reduced adherence to treatment, and increased healthcare utilization, highlighting the importance of developing competencies that support the appropriate use of health information [42].

Oral health literacy can directly influence health behaviors, particularly the use of preventive dental services. Health literacy has been identified as an important predictor of health behaviors, overall health status, and clinical outcomes [4,43,44]. Low levels of literacy are associated with poorer self-perceived health, reduced adherence to medical recommendations, limited self-care capacity, increased risk of adverse health outcomes, and higher healthcare costs [4,44,45].

Practical and public health implications

The results of the study suggest that the SMOHLQ can be a useful tool for assessing digital literacy in oral health in both research and clinical practice. Identifying the level of information literacy can facilitate the adaptation of doctor–patient communication strategies and the development of personalized educational interventions.

At the public health level, the results emphasize the need to guide patients to credible online sources and develop critical appraisal skills to reduce the impact of erroneous information distributed in the social media environment.

Study limitations

The study has some limitations that should be considered when interpreting the results. The cross-sectional design does not allow for the establishment of causal relationships between the variables analyzed, and the use of self-reporting may introduce memory or social desirability biases.

Also, the sample consisting of students may limit the generalizability of the results to the general population. Confirmatory factor analysis was not performed in the present study, which limits the possibility of statistically testing the stability of the proposed factorial structure. Although exploratory factor analysis allowed identification of latent dimensions consistent with theoretical models of digital health literacy, the factorial model cannot be considered fully confirmed. Additional studies conducted on independent samples would allow a more precise evaluation of the stability of the identified dimensions.

The high level of digital literacy characteristic of the student population may lead to an overestimation of the reported skills, limiting the extrapolation of the results to populations with different educational levels.

Data collection in a single university center may limit the generalizability of the results to other educational or cultural contexts.

Another limitation is the absence of an assessment of convergent validity by comparing SMOHLQ scores with other validated health literacy or digital literacy instruments, which could strengthen the interpretation of the results.

The study design did not include repeated measurements. Test–retest reliability was not examined, which limits the assessment of temporal consistency. Future studies should investigate the reproducibility of the questionnaire results, particularly if the instrument is intended for use in educational or clinical contexts.

The assessment of behavioral impact was based exclusively on self-report, without confirmation by objective behavioral indicators, which may introduce perception bias.

Another limitation is related to the relatively low KMO value obtained for the critical appraisal subscale, as well as the cross-loading observed for one item within the access and understanding dimension. These aspects may indicate a certain overlap between closely related competencies involved in processing online health information and suggest that minor refinement of item phrasing could further improve the factorial structure in future studies.

Strengths

In contrast to previous methodological studies that predominantly assessed internal consistency and global factor structure, the present validation explored the multidimensional structure of oral health digital literacy, highlighting distinct subscales corresponding to cognitive stages of online information processing. Strengths of the study include the adequate sample size and the comprehensive psychometric evaluation, which included factor analysis, reliability indicators, and discriminant validity assessment. The use of the ICC represents an additional methodological advantage, rarely reported in similar studies.

In addition, the approach to oral health literacy from the perspective of the social media environment reflects current trends in accessing medical information.

Implications and future directions

Future research should aim to validate the instrument in more diverse populations and in different cultural contexts. The application of confirmatory factor analysis would allow the stability of the identified structural model to be verified.

Investigating the relationship between SMOHLQ scores and objective clinical indicators could provide additional information on the real impact of digital literacy on oral health. In the future, the use of a standardized instrument could support patient-centered monitoring and the development of targeted digital educational interventions.

## 5. Conclusions

The Romanian version of the SMOHLQ demonstrated good internal consistency and a factorial structure that generally aligns with current conceptual models of digital health literacy. The findings indicate that the instrument can be used to evaluate how individuals access, interpret and apply oral health information obtained from social media sources. Further testing in more diverse populations would help clarify the stability of the identified dimensions and provide additional support for the proposed structure.

The correlations between dimensions confirm the construct validity of the questionnaire, suggesting that although information skills are interdependent, they do not always translate into behavioral changes. Overall, the SMOHLQ can be used as a valid and reliable instrument for assessing oral health literacy associated with the use of social networks, with applicability in research and in the development of educational interventions aimed at oral health. Further research on more heterogeneous populations could provide additional confirmation of the factorial structure through confirmatory factor analysis.

## Figures and Tables

**Table 1:** Objectives and indicators guiding the study design.

Objectives	Indicators	Statistical Analysis Performed
To describe the sociodemographic characteristics of the study population	Gender distribution; residence area; sample size	Frequency analysis and descriptive statistics
To evaluate the factorial suitability of SMOHLQ data	KMO index; Bartlett’s test of sphericity	Kaiser–Meyer–Olkin test and Bartlett’s test
To explore the underlying factorial structure of the questionnaire	Factor loadings; number of extracted factors; explained variance	Exploratory factor analysis (principal axis factoring with oblimin rotation)
To assess the internal consistency of SMOHLQ and its dimensions	Cronbach’s alpha coefficients	Reliability analysis (Cronbach’s alpha)
To evaluate score stability and agreement between items	Intraclass correlation coefficients (ICC)	Two-way mixed-effects ICC model
To describe the level of oral health literacy dimensions	Mean scores; standard deviation; score range	Descriptive statistics
To examine relationships between questionnaire dimensions	Correlations between Access, Critical and Behavioral scores	Pearson correlation analysis
To assess discriminant (known-groups) validity	Differences according to gender, residence area and age	One-way ANOVA

**Table 2:** Description of items corresponding to the “access and understanding” dimension.

1	I can easily summarize dental information found online.
2	I can interpret oral hygiene recommendations presented on social media.
3	I understand explanations about dental procedures presented in online videos.
4	I know which keywords to use to find relevant medical information.
5	Medical terminology used in dental posts is generally clear to me.
6	I know which social media platforms provide reliable dental information.
7	I can quickly find information about dental symptoms or treatments online.
8	Online information helps me better understand dental problems.
9	I know how to search for oral health information on social media.
10	I use social media as an initial source of information before consulting a dentist.

**Table 3:** Description of items corresponding to the “critical appraisal” dimension.

11	I check whether information is published by a medical professional
12	I analyze the credibility of the source before trusting information.
13	I can distinguish between educational content and advertising content.
14	I compare information across multiple sources before considering it correct.
15	I pay attention to scientific evidence mentioned in medical posts.
16	I question recommendations made by influencers without medical training.
17	I believe that some dental information on social media may be incorrect.
18	I can recognize exaggerated claims about rapid dental treatment results.
19	I am cautious when a post promotes “miracle” dental products.
20	I understand that the popularity of a post does not guarantee information accuracy.
21	I can identify sponsored or commercial content related to dental products.

**Table 4:** Description of items corresponding to the “behavioral impact” dimension.

22	Dental information on social media influences my oral hygiene habits.
23	I have changed how I care for my teeth based on online information.
24	I discuss information found online with my dentist.
25	Online information helps me make more informed decisions about dental treatments.
26	I feel more involved in oral health decisions due to online information.
27	I sometimes delay visiting the dentist because I first search for information on social media.

**Table 5:** Sociodemographic characteristics of the sample.

Variable	Category	N	%
Gender	Male	116	38.2
	Female	188	61.8
Residence	Urban	131	43.1
	Rural	173	56.9
Total		304	100

**Table 6:** Sampling adequacy for exploratory factor analysis.

Dimension	KMO	Bartlett χ^2^	df	*p*
Access and understanding	0.611	1392.916	45	<0.001
Critical appraisal	0.517	1958.934	55	<0.001
Behavioral impact	0.742	666.592	15	<0.001

**Table 7:** Factor loadings for the “access and understanding” dimension.

Item	Factor 1	Factor 2
I can easily summarize dental information found online	0.759	
I can interpret oral hygiene recommendations presented on social media	0.736	
I understand explanations about dental procedures presented in online videos	0.731	
I know which keywords to use to find relevant medical information	0.690	
Medical terminology used in dental posts is generally clear to me	0.624	
I know which social media platforms provide reliable dental information	0.621	
I can quickly find information about dental symptoms or treatments online	0.525	0.464
I know how to search for oral health information on social media		0.615

**Table 8:** Factor loadings for the “critical appraisal” dimension.

Item	Factor 1	Factor 2
I question recommendations made by influencers without medical training	0.956	
I analyze the credibility of the source before trusting information	0.579	
I can recognize exaggerated claims about rapid dental treatment results	0.576	
I understand that popularity does not guarantee accuracy	0.521	
I compare information across multiple sources	0.458	
I am cautious with “miracle” dental products	0.436	
I believe some dental information may be incorrect		0.826
I can identify sponsored or commercial content		0.803
I check whether information is published by a medical professional		0.486
I can distinguish educational from advertising content	0.437	0.449

**Table 9:** Factor loadings for the “behavioral impact” dimension.

Item	Factor Loading
Online information influences my oral hygiene habits	0.652
I changed how I care for my teeth based on online information	0.687
I discuss online information with my dentist	0.631
Online information helps me make informed decisions	0.704
I feel more involved in oral health decisions	0.689
I sometimes delay dental visits to search online first	0.575

**Table 10:** Reliability indices of the SMOHLQ.

Dimension	Cronbach α	ICC (Average Measures)	95% CI
Access and understanding	0.836	0.812	0.778–0.843
Critical appraisal—Factor 1	0.784		
Critical appraisal—Factor 2	0.744	0.787	0.748–0.821
Behavioral impact	0.786	0.786	0.747–0.821
Total scale	0.856	—	—

**Table 11:** Descriptive statistics of SMOHLQ dimension scores.

Dimension	N	Minimum	Maximum	Mean	SD
Access and understanding	304	3.30	4.80	4.20	0.36
Critical appraisal	304	3.55	5.00	4.54	0.40
Behavioral impact	304	1.67	4.33	2.87	0.69

**Table 12:** Pearson correlations between SMOHLQ dimensions.

Dimension	1	2	3
1. Access and understanding	1	0.603 **	0.270 **
2. Critical appraisal	0.603 **	1	0.162 *
3. Behavioral impact	0.270 **	0.162 *	1

Notes: values represent Pearson correlation coefficients (r). 1 = access and understanding; 2 = critical appraisal; 3 = behavioral impact. * *p* < 0.05, ** *p* < 0.001.

**Table 13:** Known-groups validity (ANOVA results).

Variable	Dimension	F	*p*
Gender	Access and understanding	0.406	0.524
	Critical appraisal	9.806	0.002
	Behavioral impact	3.721	0.055
Residence	Access and understanding	47.540	<0.001
	Critical appraisal	7.796	0.006
	Behavioral impact	0.371	0.543
Age	Access and understanding	21.578	<0.001
	Critical appraisal	22.281	<0.001
	Behavioral impact	7.044	0.001

**Table 14:** Mean and standard deviation of SMOHLQ scores according to gender and residence area.

Variable	Category	Access and Understanding		Critical Appraisal		Behavioral Impact	
		Mean	SD	Mean	SD	Mean	SD
Gender	Male	4.18	0.44	4.63	0.34	2.77	0.76
	Female	4.21	0.30	4.48	0.42	2.93	0.64
Residence	Urban	4.35	0.29	4.61	0.35	2.84	0.76
	Rural	4.09	0.37	4.49	0.42	2.89	0.63

## Data Availability

The original contributions presented in this study are included in the article. Further inquiries can be directed to the corresponding author.

## Abbreviations

The following abbreviations are used in this manuscript:

ANOVA	Analysis of Variance
ICC	Intraclass Correlation Coefficient
KMO	Kaiser–Meyer–Olkin Measure of Sampling Adequacy
MS	Mean Square
CI	Confidence Interval
SD	Standard Deviation
Sig	Significance
SMOHLQ	Social Media Oral Health Literacy Questionnaire
DHLI	Digital Health Literacy Instrument
